# Chemical Composition, Antioxidant Potentials, and Calcium Oxalate Anticrystallization Activity of Polyphenol and Saponin Fractions from *Argania spinosa* L. Press Cake

**DOI:** 10.3390/plants11141852

**Published:** 2022-07-15

**Authors:** Fatima Ezzahra El oumari, Naima Mammate, Hamada Imtara, Anissa Lahrichi, Radouane Elhabbani, Ghita El mouhri, Ali S. Alqahtani, Omar M. Noman, Mansour N. Ibrahim, Andriy Grafov, Dalila Bousta, Tarik Sqalli Houssaini

**Affiliations:** 1 Laboratory of Epidemiology and Research in Health Sciences, Faculty of Medicine and Pharmacy, University of Sidi Mohammed Ben Abdellah, Fez 30070, Morocco; n.mammate@usmba.ac.ma (N.M.); radouane.elhabbani@usmba.ac.ma (R.E.); Tarik.sqalli@gmail.com (T.S.H.); 2 Faculty of Arts and Sciences, Arab American University Palestine, Jenin 44862, Palestine; 3 Laboratory of Biochemistry, Faculty of Medicine and Pharmacy, University of Sidi Mohammed Ben Abdellah, BP 1893, Km 22, Road of Sidi Harazem, Fez 30070, Morocco; anissa.lahrichi@usmba.ac.ma (A.L.); ghita.elmouhri@usmba.ac.ma (G.E.m.); 4 Department of Pharmacognosy, College of Pharmacy, King Saud University, Riyadh 11451, Saudi Arabia; alalqahtani@ksu.edu.sa (A.S.A.); onoman@ksu.edu.sa (O.M.N.); 5 Department of Agricultural Engineering, College of Food and Agriculture Sciences, King Saud University, Riyadh 11451, Saudi Arabia; malsamee@ksu.edu.sa; 6 Department of Chemistry, University of Helsinki, FI-00014 Helsinki, Finland; andriy.grafov@usmba.ac.ma; 7 Morocco Laboratory of Biotechnology, Environment, Agri-Food, and Health (LBEAS), Faculty of Sciences, University of Sidi Mohammed Ben Abdellah, Fez 30070, Morocco; dalila.bousta@usmba.ac.ma; 8 Department of Nephrology, University of Hospital Hassan II, BP 1835, Atlas, Road of Sidi Harazem, Fez 30700, Morocco

**Keywords:** *Argania spinosa* L. press cake, GC–MS analysis, anticrystallization, calcium oxalate

## Abstract

A wide range of biological properties and a potent therapeutic and prophylactic effect on chronic diseases are all present in *Argania spinosa* L. press cake. The aim of this research is to valorize the anticrystallization properties against calcium oxalate crystals of *Argania spinosa* L. press cake fractions and identify its bioactive components. Chemical species identification was performed using GC–MS analysis. The turbidimetric model was used to investigate crystallization inhibition in vitro. Infrared spectroscopy technique was used to characterize the synthesized crystals. Furthermore, both DPPH and FRAP methods were used to assess antioxidant activity. The results show that the fractions are equally important in crystallization inhibition percentages of calcium oxalate crystals. For saponin and polyphenol fractions, the inhibition percentages are in the orders of 83.49% and 82.83%, respectively. The results of the antioxidant activity by DPPH method show that the two fractions are equally important in the elimination of free radicals; the inhibition percentages were 77.87 ± 4.21 and 89.92 ± 1.39 for both polyphenols and saponins, respectively. FRAP method showed that the absorbance increases proportionally with concentration, and the absorbance are almost similar for both fractions and reach maximum values in the orders of 0.52 ± 0.07 and 0.42 ± 0.03, respectively, for saponins and polyphenols. These findings demonstrate that both fractions are rich in bioactive chemicals and have an anticrystallization capacity, allowing them to be employed for the curative and prophylactic effects against urolithiasis.

## 1. Introduction

Urolithiasis, a multifactorial condition, is one of humanity’s oldest and most frequent ailments, and it continues to be a serious public health concern. It affects people of all ages, from newborns to the elderly, with a male-to-female ratio of 2:1. Around the world, a substantial number of people, between 4% and 15% of the human population, suffer urinary stone problems [1]. During the first 5 years, the recurrence rate has been estimated to be 50%, with calcium oxalate being the most common cause [2,3,4]. Nucleation, crystal development, crystal aggregation, and crystal retention are all steps in the multi-step process of urinary stone pathogenesis [5]. Stone formation requires supersaturated urine. Supersaturation is influenced by various factors such as urinary pH, ionic strength, solute concentration, and complexations [5,6].

Modern urology practice has been revotionlized by the adoption of kidney stone treatment techniques as extracorporeal shock wave lithotripsy, ureteroscopy, and percutaneous nephrolithotomy. However, kidney stones recurrence is unaffected [7,8]. Renal impairment, severe hematuria, pancreatitis, infection, and persistent residual fragments as probable nidus for new stone formation are a few of the side effects of these treatments, in addition to its high cast and recurrence problems [9,10]. Despite significant advancements in the pathogenesis and treatment of urolithiasis, no suitable medication is currently being utilized in clinical practice. As a result, a medicine that could prevent the condition or its recurrence would be quite attractive. Phytochemicals have been used to conduct herbal therapy [11]. It’is now acknowledged by the World Health Organization (WHO) as an essential building block for basic health care around the world. Herbal medicines are known to include multiple substances that act on urolithiasis via multiple mechanisms [12]. Several biological effects of polyphenols have been achieved, such as antioxidant and antimicrobial activities [13,14]. The antiurolithiatic potential of a polyphenol-rich extract from *Quercus gilva* Blume was linked to its antioxidant and anti-inflammatory activities [15]. Furthermore, grape seed polyphenols protected the renal papilla from calcium monohydrate oxalate-induced calculi development in [16]. The saponin-rich fraction made from *Solanum xanthocarpum* Schrad. and Wendl., *Herniaria hirsuta* L., and hydroalcoholic extract from *Argania spinosa* press cake inhibited in vitro calcium oxalate crystal nucleation in artificial urine solution [14,15,16,17,18,19].

Sapotaceae family comprises the endemic tree *Argania spinosa* L. of southwestern Morocco [20]. The oil produced from the fruits of *Argania spinosa* L. significantly influences the socioeconomic well-being of Amazigh women. There are 2 to 3 kernels in each argan fruit which utilized to make argan oil. Oil made from unroasted kernels is used in cosmetics, while roasted kernels are used to make culinary oil. *Argania spinosa* L. press cake resulting from unroasted kernels is a rich source of bioactive phytochemicals (polyphenols and saponins) [21].

Several studies have revealed many pharmacological effects of *Argania spinosa* L., such as the curative and preventive potential of the plant against chronic diseases, as well as its numerous biological properties, which include antibacterial, antioxidant, anti-hyperglycemic, anti-tumor, anti-proliferative, and insecticidal [18,19,20]. Other studies have highlighted the presence of several phytochemicals, such as catechol, tyrosol, catechin, and epicatechin [21]. In addition, several pharmacological activities of *Argania spinosa* L. press cake have been confirmed [22,23,24]. The usefulness of these two fractions against kidney stone development and the phytochemical composition of each fraction have not yet been verified by research. Using the anticrystallization approach, the current study’s objective was to identify the chemical components present in the poly-phenol and saponin fractions of *Argania spinosa* L. Skeels press cake and assess their contribution to antiurolithiatic activity.

## 2. Results

### 2.1. GC–MS Analysis

**Figure 1:**
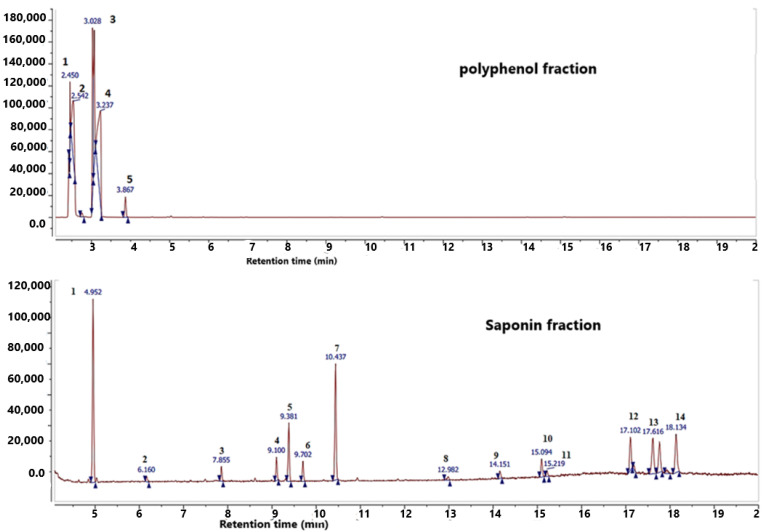
Chromatographic profile of polyphenol and saponin fractions extracted from *Argania spinosa* L. press cake.

By comparing the GC-MS absorbance and MS spectra with data from the literature, it was possible to determine biochemicals of the polyphenol and saponin fractions of the *Argania spinosa* L. press cake. The identification outcomes and chromatographic profiles for these two fractions are displayed in Figure 1 and Table 1 and Table 2. The examined fractions contained many substances that were confirmed by GC-MS analysis, notably isocyanic acid, isobutyric acid, p-hydroxybenzoic acid, catechol, and ephedrine in polyphenol fraction, and beta-D-galactofuranose,1,2,3,5,6-pentakis-o-(trimethylsilyl), D-xylofuranose,1,2,3,5 tetrakis-o-(trimethylsilyl), glucofuranoside, methyl-tetrakis-o-trimethylsilyl, and D-ribopyranose,1,2,3,5 tetrakis-o-(trimethylsily) in saponin fraction.

### 2.2. Antioxidant Activity

**Figure 2:**
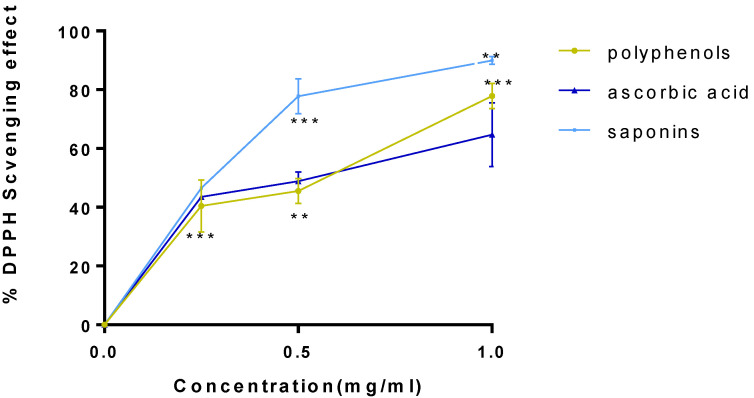
Percent inhibition of free DPPH by saponin and polyphenol fractions. ** *p* < 0.01; *** *p* < 0.005 vs. ascorbic acid.

**Figure 3:**
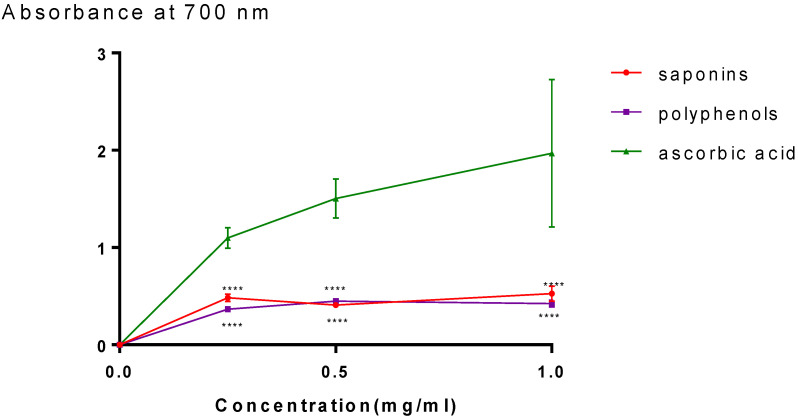
Ferric reducing power of saponin and polyphenol fractions; **** *p* < 0.005 vs. ascorbic acid.

DPPH and FRAP techniques were used to test the antioxidant activity. To assess the significance of the antiradical effect of the saponin and polyphenol fractions, Ascorbic acid was utilized as an antioxidant’s positive control sample. In fact, Figure 2 and Figure 3 show the percent inhibition and the median inhibitory concentration (IC_50_). The inhibition rate for the three products increases rapidly as concentration rises, as shown in Figure 2, which illustrates the DPPH radical’s reducing activity. The maximum inhibitory percentages are equivalent to 89.92 ± 1.39 percent at a concentration of 1 mg/mL of saponin fraction (*p* < 0.05 vs. ascorbic acid). For polyphenol fraction, the maximum inhibition percentage corresponding to the maximal concentration (1 mg/mL) was 77.87 ± 4.21 (*p* < 0.005 vs. ascorbic acid), against 64.72 ± 5.82 for the positive control.

These results demonstrate that low doses of plant fractions can effectively remove free radicals by converting them into more stable molecules. In reality, offering a free radical hydrogen transforms it into a nonreactive species. The strange electron characteristic, which is the cause of radical reactivity, is eliminated by the addition of hydrogen.

The Fe^3+^/ferricyanide complex can be reduced to a ferrous Fe^2+^ form thanks to the two fractions’ high ferric reducing antioxidant power (FRAP), which shows a high capacity for electron donation. Figure 3 demonstrates the correlation between the absorbance and concentration. The values are nearly identical for both fractions and reach maximum values in the orders of 0.52 ± 0.07 (*p* < 0.005 vs. ascorbic acid) and 0.42 ± 0.03 (*p* < 0.005 vs. ascorbic acid) respectively, for saponins and polyphenols. The absorbance for ascorbic acid rises quickly and reaches a value of 1.96 ± 0.75.

### 2.3. In Vitro Calcium Oxalate Crystallization Assay

**Figure 4:**
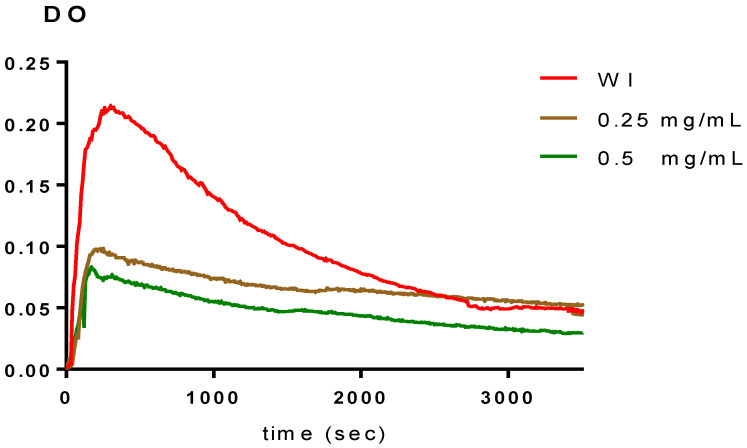
Temporal variation of the optical density in the presence of polyphenol fraction.

**Figure 5:**
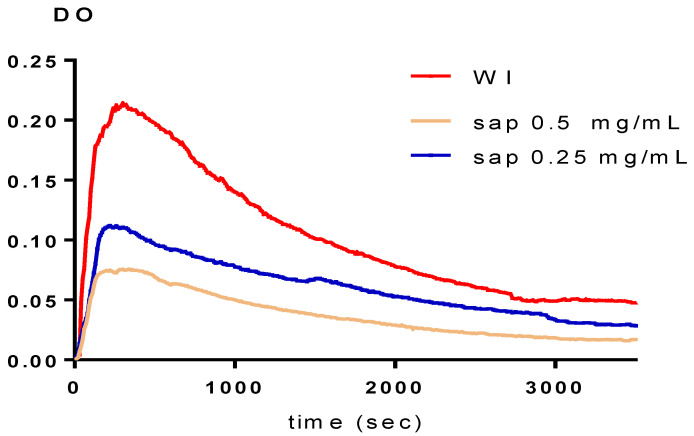
Temporal variation of the optical density in the presence of saponin fraction.

**Figure 6:**
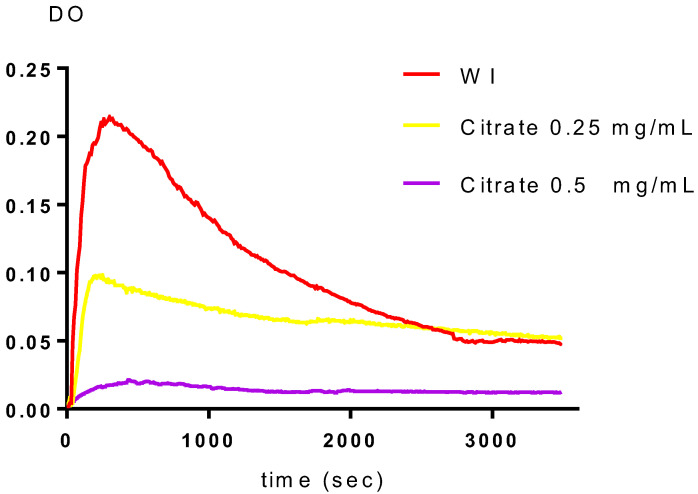
Temporal variation of the optical density in the presence of potassium citrate. WI: without inhibitor.

In vitro experiments using the turbidimetric model have been conducted in both the absence (WI) and presence of inhibitors. The percentages of inhibition were calculated by contrasting the slopes with and without the inhibitors, and the findings are displayed in Table 3 and Figure 4, Figure 5 and Figure 6. According to Table 3, the two fractions significantly reduce the growth of calcium oxalate crystals. Both the polyphenol and saponin fractions had approximately a similar percentage inhibition of nucleation. Indeed, they are 79.76 ± 5.4% to 82.83 ± 4.32% for concentrations from 0.25 to 0.5 g/L and 78.87 ± 4.03% to 83.49 ± 3.73% for concentrations from 0.25 and 0.5 g/L, with R > 0.97 and CV decreased by 10% for both (*p* < 0.005 for all comparisons vs. Cit.K). The inhibition percentage of citrate potassium, however, is marginally higher, ranging from 75.47 ± 6.76% to 97.28 ± 0.29% (*p* < 0.005 for all comparisons vs. Cit.K) for concentrations of 0.25 and 0.5 g/L, with R > 0.98 and CV less than 10%. Figure 4, Figure 5 and Figure 6 illustrate the curves that indicate how concentration affects the nucleation inhibition rate in the Cit.K solution and both fractions, respectively.

### 2.4. Characterization of Crystals by Fourier-Transform Infrared Spectroscopy

**Figure 7:**
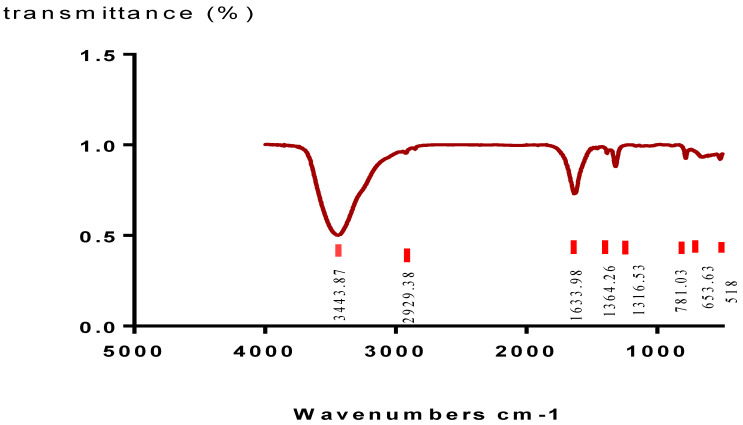
Infra−red spectrum of Ca−Ox synthesized crystals.

The chemical composition of the synthesized crystals was determined using infrared spectroscopy technique (FTIR). The resulting spectra are shown in Figure 7. Two bands, 3443 and 2929 cm^−^^1^, which are critical to the OH stretching of the water, were found in the spectra after analysis. An out-of-plane C-O deformed band and then another O-C-O plane bending have been identified at 781 and 518 cm^−^^1^, respectively. The stretching bands of antisymmetric carbonyls (vas (COO)) and symmetric metal carboxylates (vs. (COO)) are both compatible with the absorption bands at 1633 cm^−^^1^ and 1364/1318 cm^−^^1^, respectively; these bands belong to COT crystals [25].

## 3. Discussion

Current pharmaceuticals require the development of natural products based on therapeutic agents in order to treat or prevent diseases. A non-exhaustive source of potentially bioactive compounds has long been recognized in medicinal plants, and they remain an important source for the development of modern therapeutic options [26].

The aim of our study is to valorize and verify the efficacy of polyphenol and saponin fractions extracted from *Argania spinosa* L. press cake in order to reduce or inhibit calcium oxalate crystallization. We can determined the nucleation phase and the induction time using the turbidity model, which is based on the measurement of the absorbance in solutions as a function of time. The latter is still an important factor because it predicts when nucleation will start. The induction time in this study is dependent on the concentration. This finding explains why the inhibitory impact of fractions causes a delay in crystal formation. Additionally, the polyphenol and saponin fractions are even effective, as shown by a comparison of the induction time values at varied concentrations. Additionally, our results concur with those of Hess et al. [27] and Driouch et al. [28]. The level of nucleation inhibition is quite high in both fractions, with little variation in concentrations examined. These outcomes vastly outweigh those of S. H. Youn et al. and Grases et al., who worked on saponin fractions and polyphenol [15,16]. The efficacy of positive control (potassium citrate) on inhibiting the nucleation step becomes more important at a concentration of 0.5 mg/mL. The assessment of the nucleation rate is useful for estimating the lithogenic potential and assessing the efficacy of therapeutic approaches aimed at reducing the probability of recurrence.

For the evaluation of antioxidant activity, the role of an antioxidant is to remove free radicals. In vitro antioxidant activities of DPPH and FRAP were used to test the antioxidant power of saponin and polyphenol fractions. For natural compounds, the DPPH assay is a popular radical scavenging test. The antioxidant caused a decrease in the DPPH radical’s absorption generated by hydrogen-donating organisms scavenging the radical. Our outcomes revealed that both fractions (saponin and polyphenol) exhibit an antioxidant activity with the maximum inhibition percentages in the orders of 89.92 ± 1.39% and 77.87 ± 4.21%, respectively. Superoxide anions can be catalyzed and induce more dangerous hydroxyl radicals by ferrous ions (Fe^2+^). The reducing power of plant extracts is commonly used to assess their antioxidant activity [29,30,31]. Our in vitro antioxidant study showed that both fractions have similar antioxidant with maximum values in the orders of 0.52 ± 0.07 and 0.42 ± 0.03, respectively, for saponins and polyphenols.

The chromatographic analysis revealed the existence of various components in both fractions of *Argania spinosa* L. press cake that may be implicated in antiurolithiatic activity against calcium oxalate crystals. A total of 19 substances were discovered by GC–MS analysis in this study, including isobutyric acid, p-hydroxybenzoic acid, catechol, ephedrine, oleic acid, and morphinan-6-one. Some of the chemicals found in *Argania spinosa* L. press cake (L) extracts have been previously confirmed to have antioxidant, anti-inflammatory, antihyperglycemic, and antiproliferative properties [16,18,19,20].

The anti-inflammatory, antimutagenic, antiviral, antimicrobial, hypoglycemic, estrogenic, and antioxidant properties of p-hydroxybenzoic acid have been confirmed; moreover, it decrease the probability of developing hypertension [28,32,33]. Isobutyric acid (2-methylpropanoic acid) has been discovered to have antiproliferative effect on CFSC-2C hepatic stellate cells [34]. Ephedrine has a positive ionotropic effect, hence the increase in blood pressure [35]. Beta-D-glucopyranose is confirmed to have an anticancer potential [36]. Additionally, oleic acid has anti-inflammatory properties and has a function in the activation of many immunological pathways in immune competent cells [37]. Analgesic properties were determined for morphinan-6-one [38]. Our result of GC–MS analysis revealed some substances, which are identified by Charrouf et al. [39], such as catechol, oleic acid, p-hydroxybenzoic acid, and beta D-glucopyranose. This study’s findings about the antiurolithiatic effect on calcium oxalate crystals’ capacities may be explained by the abundance of several chemical compounds with a variety of activities, as documented in the prior literature.

## 4. Materials and Methods

### 4.1. Plant Material

In July 2019, the fruits of *Argania spinosa* L. were collected in the Agadir region of Morocco. Before use, the fruits were peeled and pulped. To obtain kernels, nuts were cracked, and each kernel was mechanically crushed. Press cake from *Argania spinosa* L. was dried at 25 °C before being ground into a fine powder.

### 4.2. Extraction Methods

#### 4.2.1. Polyphenols Extraction Method

Following the method desrcibed by Mahmoudi et al. [40], polyphenols were extracted. 40 mL of the extraction solvent (ethanol, acetone, and methanol 70 percent *v*/*v* in water) was added along with 1 to 2 g of the plant powder. Before filtering, each combination of solutions was heated in a water bath for 30 min. After being removed, the marcs were filtered. The combined filtrates were stored at 4 °C until use after being centrifuged at 4000 rpm for 20 min.

#### 4.2.2. Saponin Extraction Method

According to the steps mentioned by Majinda et al. [41], saponins were extracted with an ethanol solvent from powdered plant material. After that, a defatting step (to remove lipophilic compounds) with petroleum ether was performed after the extraction process. After removing the lipophilic compounds, the extracts were shaken with an n-butanol solvent. After combining the n-butanol aliquots and removing the solvent, a crude saponin extract was obtained.

### 4.3. GC–MS Analysis

According to Kabran’s approach [42], the phytochemical components were identified using the silylation method. Separated, dried on anhydrous MgSO_4_, and then concentrated under vacuum, the organic fractions were processed. Then, after being heated at 37 °C for 30 min, 3 mg of the resulting fraction was combined with 200 L of N-methyl-N-trimethylsilyltrifluoroacetamide (MSTFA). The samples were then analyzed using a gas chromatograph connected to a mass spectrophotometer (GC-MS; Agilent Technologies Model 5973 with an Agilent column 19091S-433 HP-5MS equipped with a single quadrupole mass spectrometer, operated using electron ionization (EI)) to determine the composition of 0.1 L of the samples (Agilent Technolo-gies, Santa Clara, CA, USA). Helium served as the carrier gas and had an average pressure range (psi) of 0.9 mL/s. The oven was set to a temperature range of 60 to 300 °C. The retention time of the silylated compounds was compared with those of the standards acquired from the database to identify them.

### 4.4. Antioxidant Activity

#### 4.4.1. DPPH Method

With a few minor changes of Oktay et al. protocol [29], the DPPH (1,1-diphenyl-2-picrylhydrazyl) technique was used to measure the antiradical activity of both fractions in triplicate. In fact, a DPPH solution was created by dissolving 4 mg of DPPH in 100 mL of methanol. Ascorbic acid (vitamin C), at concentrations of 0.25 to 1 mg/mL, and 750 mL of fractions with concentrations ranging from 0.1 to 2 mg/mL were added to test tubes. After 30 min of incubation in the dark at room temperature, the absorbance (Ab) was measured at a wavelength of 517 nm (Labtron Equipment Ltd., Camberley, UK). The percentages of inhibition were estimated using the following formula:

% scavenging radicals = ((Ab control − Ab simple)/Ab control) × 100.

#### 4.4.2. FRAP Method

Raman method [30] was used to determine reduction power of the plant fractions. Combining 2.5 mL of potassium ferricyanide [K_3_Fe (CN)_6_], 2.5 mL of phosphate buffer (0.2 mol/L, pH 6.6), and 2.5 mL of polyphenol and saponin fractions solubilized in destilled water at various concentrations ranging from 0.25 to 1 mg/mL is required. The combination was then incubated for a further 20 min at 50 °C. The mixture was mixed with 2.5 mL of 10% trichloroacetic acid, and then centrifuged at 3000 rpm for 10 min. 2.5 mL of the solution’s top layer, 2.5 mL of distilled water, and 0.5 mL of FeCl_3_ were mixed (0.1 percent) The absorbance was measured in triplicate at 700 nm (Labtron Equipment Ltd., Camberley, UK). Ascorbic acid was used as a control [26,27].

### 4.5. In Vitro Calcium Oxalate Crystallization Assay

The Hess et al. approach [27] was used to examine the calcium oxalate anticrystallization impact of saponin and polyphenol fractions. Stock solutions of CaCl_2_:2H_2_O (15 mM) and Na_2_C_2_O_4_ (1.5 mM) containing 200 mM NaCl were made, and 10 mM sodium acetate was added to bring the pH to 5.7. The solutions were then warmed to 37 °C and used after being filtered through 0.22 m pore diameter filters. For crystallization studies without an inhibitor, 1 mL of CaCl_2_: 2H_2_O solution and 1 mL of distilled water were transferred to a quartz cuvette with an optical path of 10 mm. Next, an identical amount of Na_2_C_2_O_4_ solution was added to initiate the crystal’s development. The optical density (OD) was measured using a UV-visible spectrophotometer (Labtron Equipment Ltd., Camberley, UK) at a wavelength of 620 nm every 15 s for 58 min. In a manner similar to the tests without inhibitors, the extracts were used in place of distilled water to conduct the studies with inhibitors at varied concentrations of 0.25 and 0.5 g/L of fractions. Additionally, the slopes of the nucleation phase “SN” were determined using linear regression analysis, and the percent-ages of inhibitions were obtained using the Hess et al. relation [27].

### 4.6. Characterization of Crystals by Fourier-Transform Infrared Spectroscopy

Infrared spectroscopy technique (FTIR) was used to characterize the crystals between 4000 and 400 cm^−1^ with a resolution of 4 cm^−1^ (Bruker Optics GmBH & Co. KG, Ettlingen, Germany). In practice, a volume V of a CaCl_2_: 2H_2_O (15 mM) solution received the equivalent volume of inhibitor and Na_2_C_2_O_4_ (1.5 mM) solution. After being stirred constantly for 30 min, the solution was allowed to stand for 1 h at 37 °C. The crystals were then dried, centrifuged three times at 4000 rpm, and washed three times with ethanol.

### 4.7. Data Analysis

Mean ± SD was used to express the results. One-way ANOVA was used for the statistical analysis, which was then followed by Tukey’s multiple comparison test and Pearson’s correlation using GraphPad Prism 7. *p* < 0.05 was considered significant.

## 5. Conclusions

The use of medicinal and aromatic herbs in the urolithiatic population for urolithiasis prevention and treatment especially in cases of recurrence, a serious public health issueremains an alternative to medical techniques. The purpose of this study is to experimentally confirm the effectiveness of the polyphenol and saponin fractions of *Argania spinosa* L. press cake in inhibiting the crystallization of calcium oxalate. Our findings show that polyphenols and saponins had nucleation inhibition rates of 82.83% and 83.49%, respectively. In comparison with potassium citrate, we found that both fractions have a similar efficiency and potassium citrate has a slightly higher efficiency. However, in vivo investigations on animal models are required in order to assess the fraction potential medicinal efficacy.

## Figures and Tables

**Table 1:** Chemical compounds identified in polyphenol fraction.

No.	Retention Time (Min)	Compounds	Chemical Formula
1	2.43	Isocyanic acid/carbimide hydrogen isocyanate	HNCO
2	2.75	Propanoic acid,2-methyl-[cas] (isobutyric acid)	C_4_H_8_O_2_
3	3.03	p-Hydroxybenzoic acid	C_7_H_6_O_3_
4	3.23	Catechol	C_6_H_6_O_2_
5	3.87	Ephedrine	C_10_H_15_NO

**Table 2:** Chemical compounds identified in saponin fraction.

No.	Retention Time (Min)	Compounds	Chemical Formula
1	4.96	Silanamine, N,1,1,1-tetramethyl-N-(trimethylsilyl)	SiH_5_N
2	7.85	3-Methyl-6-phenyl-imidazole [2,1-b] oxazole	C_13_H_10_N_2_O_2_S
3	9.1	3,7-Dioxa-2,8disilanonane,2,2,8,8-tetramethyl-5-(trimethylsilyl)oxypropoxy)silane)	C_9_H_24_O_2_Si_2_
4	9.38	N-methyl-n-phenyl-n’-(3-methoxyphenyl)-urea	C_15_H_16_N_2_O
5	9.7	Morphinan-6-one, 3-methoxy-17-methyl	C_18_H_21_NO_3_
6	10.43	Imidazole,1,4-dimethyl-2-phenyl	C_23_H_20_N_2_
7	14.15	Quinidine	C_20_H_24_N_2_O_2_
8	15.09	Oleic acid TMS	C_18_H_34_O_2_
9	15.21	Tetramethoxyflavone	C_19_H_18_O_6_
10	17.1	Beta-D-galactofuranose,1,2,3,5,6-pentakis-o-[trimethylsilyl]	C_21_H_52_O_6_Si_5_
11	17.61	D-xylofuranose,1,2,3,5 tetrakis-o-[trimethylsilyl]	C_17_H_42_O_5_Si_4_
12	18.13	Glucofuranoside, methyl-tetrakis-o-trimethylsilyl	C_19_H_46_O_6_Si_4_
13	17.78	D-ribopyranose,1,2,3,5 tetrakis-o-[trimethylsilyl]	C_17_H_42_O_5_Si_4_

**Table 3:** Percent inhibition of nucleation in the presence of polyphenol and saponin fractions and potassium citrate.

Concentration g/L	% Inhibition			R			CV		
	P.F	S.F	Cit.P	P.F	S.F	Cit.P	P.F	S.F	Cit.P
0.25	79.76 ± 5.4	78.87 ± 4.03	75.47 ± 6.76	0.96	0.97	0.99	6.77	4.46	8.96
0.5	82.83 ± 4.32	83.49 ± 3.73	97.28 ± 0.29	0.97	0.97	0.97	5.21	5.11	0.3

Values are expressed as mean ± standard deviation (*n* = 3). *p* < 0.05; P.F: polyphenol fraction; S.F: saponin fraction; Cit.P: potassium Citrate; R: correlation coefficient; CV: variation coefficient.

## Data Availability

Not applicable.
